# The outbreak of rabbit hemorrhagic virus type 2 in the interior of China may be related to imported semen

**DOI:** 10.1016/j.virs.2022.04.003

**Published:** 2022-05-02

**Authors:** Ruibin Qi, Chunchun Meng, Jie Zhu, Hang Li, Qiuhong Miao, Jingyu Tang, Aoxing Tang, Hongyuan Guo, Chuncao Liu, Chuanfeng Li, Zongyan Chen, Fang Wang, Qinwen Zhang, Guangqing Liu

**Affiliations:** a Innovation Team of Small Animal Infectious Disease, Shanghai Veterinary Research Institute, Chinese Academy of Agricultural Sciences, Shanghai, 200241, China; b Institute of Veterinary Medicine, Jiangsu Academy of Agricultural Sciences, Nanjing, 210014, China; c Veterinary Medicine Department of Agricultural and Animal College, Qinghai University, Xining, 810016, China

## Abstract

• We identified one RHD case caused by a new RHDV variant (GI.2) in China through HA, TEM, and genome sequencing.
• This is the first study to demonstrate that GI.2 can replicate efficiently in the reproductive system.
• Our evidence suggests that GI.2 might be introduced into China by contaminated rabbit semen.

Dear Editor,

Rabbit hemorrhagic disease (RHD) is a fatal infectious disease that primarily affects adult rabbits and causes great economic losses in the rabbit industry (Mitro and Krauss, 1993). The causative pathogen is rabbit hemorrhagic disease virus (RHDV), which belongs to the genus *Lagovirus* and the family *Calicivirus* (Moussa et al., 1992). In 1984, RHDV was first documented in China; it was found in a group of commercially bred *Angora* rabbits imported from Germany. Whether those rabbits were infected with RHDV before importation from Germany is difficult to clarify. One study suggests that RHDV has spread across Europe for many years prior to 1984 (Kerr et al., 2009).

All RHDV strains are classified into two different serotypes, and several different subtypes have been identified, including classic RHDV (GI.1/G1–6) and RHDV2 (GI.2). GI.2 is a new variant of RHDV that was first reported in northwestern France in 2010 (Abrantes et al., 2012). It has since spread across parts of Europe, Australia, north America, and Africa (Abrantes et al., 2013; Hall et al., 2015; Martin-Alonso et al., 2016; Rouco et al., 2019, 2020). Compared with GI.1, GI.2 is hosted in a wider range of species, not only domestic rabbits but also hares, including European hares (*L. europaeus*), Sardinian cape hares (*L. capensis*), Corsican hares (*L. corsicanus*), and jack rabbits in the USA (Puggioni et al., 2013; Camarda et al., 2014; Neimanis A. S. et al., 2018). Notably, GI.2 can have a high mortality rate (70%–100%) in young rabbits, which has never happened in GI.1 outbreaks. Moreover, the GI.1 vaccine in immunized rabbits showed insufficient protection against GI.2 strains (Le Gall-Reculé et al., 2011).

Although China is the largest rabbit breeder in the world, no GI.2 strain has been isolated in China. However, in May 2020, a GI.2 outbreak occurred in Sichuan Province, a typical inland area of China, and killed thousands of young rabbits. In this study, we described the epidemic features and genomic characterization of the representative isolate (SC20-01), and we analyzed the potential transmission source of the pathogen.

Numerous rabbit deaths were reported at two rabbit farms near Chengdu, a city in the interior of China. Among those rabbits, all adult females were immunized with RHDV and inactivated Pasteurellosis Multocida Propolis-adjuvant vaccine (Strain YT ​+ ​Strain JN), and all young rabbits were unimmunized. Most of the dead rabbits were young (less than 30 days old), and the death rate was approximately over 40%. However, mortality among adult female rabbits was less than 5%. Moreover, all the dead rabbits showed typical clinical signs of RHD, including anorexia, opisthosomas, and bloody nasal discharge. The major autopsy findings were hemorrhages and congestion in the lungs, heart, and kidneys, as well as acute hepatitis and splenomegaly.

To identify the causative pathogens, the livers of the dead rabbits were homogenized and centrifuged for supernatant collection. A hemagglutination assay (HA) test was performed using these supernatants and 1% human erythrocytes of blood group O (Liu et al., 1984). The supernatants were found to significantly agglutinate human type O erythrocytes, and the HA titer was 1:800. Subsequently, the supernatants were clarified with 30% sucrose and collected for analysis with transmission electron microscopy (TEM), which revealed icosahedral symmetry viral particles with a 30–40 nm diameter that were consistent with those of RHDV (Fig. 1A).

**Fig. 1:**
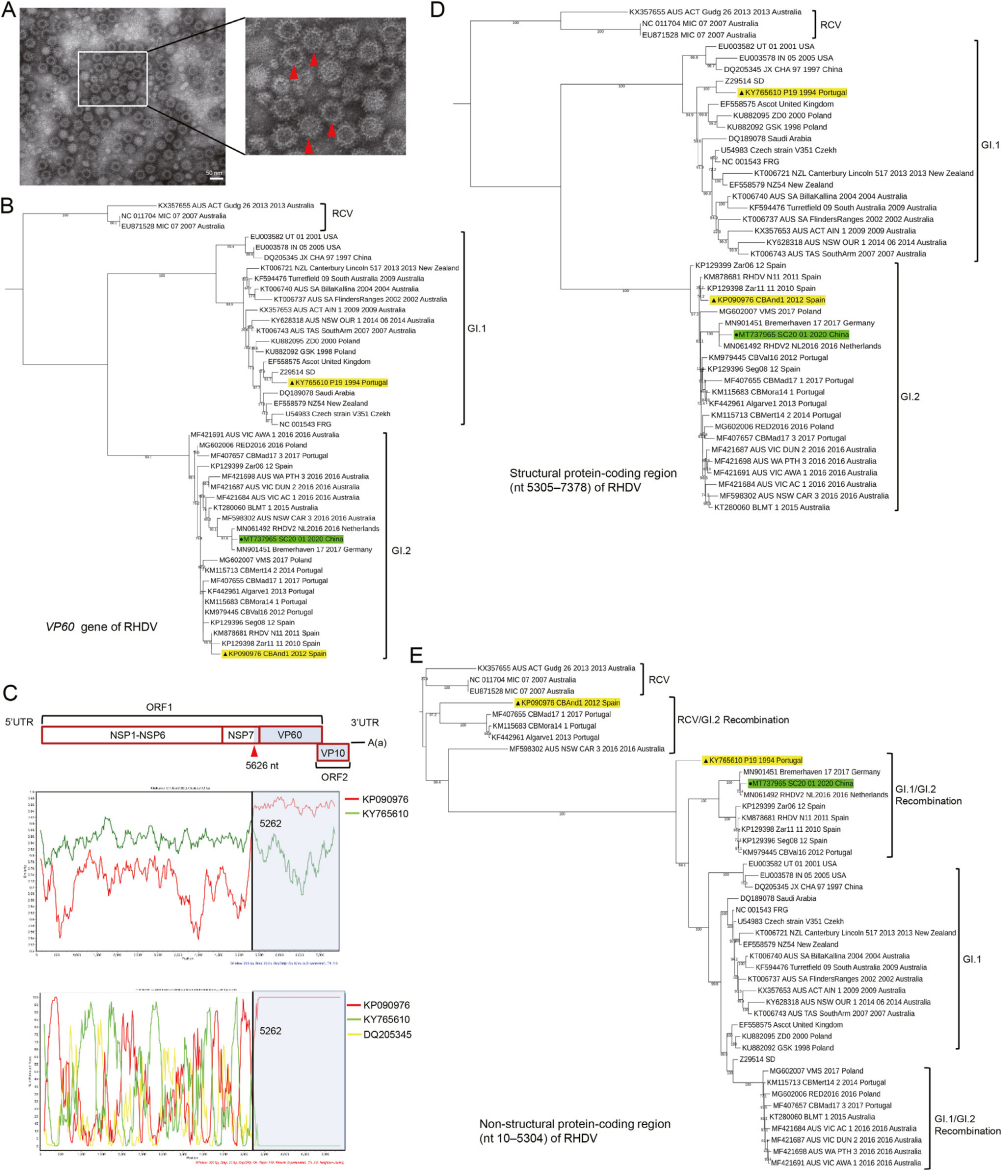
EM images and phylogenetic analysis of the GI.2 SC20-01 strain. **A** EM images of viral particles extracted from the supernatants of the homogenized livers of infected rabbits. The supernatants of the homogenized livers were clarified with a 30% sucrose solution. Icosahedral symmetry viral particles were observed with diameters ranging from 30 to 40 nm, which was consistent with those of RHDV (red arrows). **B***VP60* gene sequences of RHDV were inferred through the maximum likelihood method with 1000 replicates using MEGA5.0 software. **C** Similarity plot and results of bootscanning analyses of RHDV SC20-01 (MT737965) based on the full-length genome. CBAnd1 (KP090976) and P19 (KY765610) were used as putative parental strains, and JX/CHA/97 (DQ205345) was used as the control strain. A sliding window of 200 nucleotides, moving in 20-nucleotide steps, was used in this analysis. The X-axis indicates the percentage of similarity or permuted trees, and the Y-axis indicates the position of the full-length genome of RHDV. The genome of SC20-01 was divided by single break point into two regions, and the recombinant site was labeled. Structural protein-coding region (nt 5305–7378) (**D**) and non-structural protein-coding region (nt 10–5304) (**E**) sequences of RHDV were inferred through the maximum likelihood method with 1000 replicates using MEGA5.0 software. Black triangles with yellow labels indicates the recombination parental strains. Black dot with green label indicate the isolation strain.

To confirm causative pathogens through sequencing, total RNA from the livers of dead rabbits was extracted using TRIzol Reagent (Invitrogen, Boston, USA) and reverse transcribed using the M-MLV reverse transcriptase (Promega, Madison, USA). Primers, which are used to amplify the full-length genome sequence of those isolates, were designed based on the sequence of the JX/CHA/97 strain (DQ205345) (Liu. et al., 2006). The PCR products were cloned into pMD18-T and sequenced. Although the case of GI.2 occurred on two separate farms, the distance between them was less than 5 km. We collected samples from both farms; when returning to the laboratory, we found that the genome sequences were completely consistent. Therefore, only one representative strain (SC20-01) was selected for the following analyses. The complete nucleotide sequence of SC20-01 consisted of 7,435 nt (excluding the poly [A] tail) and contained two open reading frames (ORF1 and ORF2). The complete sequence of SC20-01 has been submitted to GenBank (accession no. MT737965).

To analyze the genomic characterization of the SC20-01 strain, 19 and 21 representative genome sequences of the GI.1 and GI.2 endemic strains, respectively, from Europe and Australia were selected randomly and downloaded from GenBank (Supplementary Table S1). Additionally, three genome sequences of rabbit calicivirus (RCV) were chosen as the out-groups. A phylogenetic tree was constructed using the maximum likelihood statistical method based on the *VP60* gene using MEGA5.0 software (www.megasoftware.net). Based on the phylogenetic tree, the SC20-01 strain was most closely related to the RHDV2-NL2016 strain (accession no. MN061492) and the Bremerhaven-17 strain (accession no. MN901451), which were all isolated from rabbits in Europe (Fig. 1B). The homology of ORF1 between SC20–01 and RHDV2-NL2016 was 99.2%, while ORF2 was completely consistent. This suggests that SC20-01 might have originated in Europe.

There are many genetic recombination events among RHDVs; furthermore, the inter-subtype genetic recombination events are mainly located in the ORF1s (Qi et al., 2019). After selecting 324 genome sequences of RHDV and three genome sequences of RCV from GenBank, we chose two strains (P19 [Portugal, accession no. KY765610, GI.1b] and CBAnd1 [Spain, accession no. KP090976, GI.2]) as the putative parental strains to analyze the recombination events in the SC20-01 strain using the recombination detection program (RDP) v4.56 software with different algorithms (GENECONV, Bootscan, RDP, Maximum Chi-Square, Chimaera, Sister Scanning, and 3seq). The recombinant region of the SC20-01 strain was found to be located in the ORF1 region (nucleotide 5248 to 5387). Furthermore, a recombination event of the SC20-01 strain was confirmed using SimPlot with the observation of an apparent breakpoint (5262 nt in *NSP7* of *ORF1*) that separated the SC20-01 genome into two regions, which was consistent with the RDP results (Fig. 1C). This recombination event was identical to that of RHDV2-NL2016, which further indicated that the two strains have the same origin. According to the results of a genetic algorithm for recombination detection (GARD, www.datamonkey.org), three recombination sites (nt 153, 924, and 5262) were detected, with the model averaged support higher than 50%. Combined with the RDP results, the recombination site nt 5262 is located in the recombination region nt 5248–5387, which is consistent with the result of Simplot. The most reliable recombination site is nt 5262. Further, phylogenetic trees were constructed based on structural (nt 5305–7378, Fig. 1D) and non-structural (nt 10–5304, Fig. 1E) protein-coding regions of the SC20-01 strain with MEGA5.0 software using the maximum likelihood statistical method (www.megasoftware.net). The non-structural protein-coding region of SC20-01 was more closely related to that of GI.1 than that of GI.2. This also strongly demonstrated that the SC20-01 strain was a GI.1b/GI.2 recombination strain.

To verify the pathogen in this outbreak, six 6-week-old female specific pathogen free (SPF) New Zealand white rabbits (*Oryctolagus cuniculus*) were randomly divided into two groups of three and intramuscularly injected with 1 mL of the supernatants of homogenized livers or phosphate buffer saline (PBS); then, they were observed for three days. After 24 hours post-infection, two rabbits in the experimental group showed typical clinical symptoms of RHD, and one died. The two surviving rabbits in the experimental group were euthanized using an intravenous injection of sodium pentobarbital to examine the tissue distribution of pathogens at various post-infection stages. After observation for seven days, all rabbits in the control group survived and did not show any clinical RHD symptoms. All surviving rabbits were euthanized with an intravenous injection of sodium pentobarbital 14 days after infection.

To examine the viral distribution, total RNA in the tissue and body fluid of rabbits infected with the SC20-01 strain was extracted (Table 1). A SYBR-Green I-based real-time quantitative polymerase chain reaction with the primers targeting the conserved GI.2 *VP60* region was used to detect viral loads (*VP60* gene copies per μg of total RNA). Among the tissues and body fluids, there was a higher viral load in the liver, cholecyst, spleen, and lung, which was consistent with previous findings (Liu et al., 2015; Neimanis A. et al., 2018). Furthermore, there was a high viral load in the gonads and serums of the infected rabbits.

**Table 1:** Detection of viral loads in tissues and body fluids of rabbits infected with SC20-01.

Tissue^a^	Unobvious symptoms	Obvious symptoms	Dead
Liver	3.80 ​× ​10^7^	9.19 ​× ​10^7^	2.82 ​× ​10^7^
Cholecyst	6.37 ​× ​10^7^	9.27 ​× ​10^7^	2.99 ​× ​10^7^
Lung	5.11 ​× ​10^7^	1.89 ​× ​10^6^	1.64 ​× ​10^7^
Spleen	4.05 ​× ​10^7^	4.47 ​× ​10^7^	3.06 ​× ​10^7^
Heart	5.47 ​× ​10^3^	4.66 ​× ​10^4^	7.09 ​× ​10^5^
Kidney	5.98 ​× ​10^4^	2.53 ​× ​10^6^	7.80 ​× ​10^6^
Gonad	1.52 ​× ​10^5^	1.92 ​× ​10^5^	2.83 ​× ​10^6^
Urine	2.96 ​× ​10^2^	1.01 ​× ​10^3^	n.c.
Pancreas	n.c.^b^	4.53 ​× ​10^6^	6.78 ​× ​10^6^
Brain	2.35 ​× ​10^4^	1.67 ​× ​10^7^	4.12 ​× ​10^5^
Trachea	1.81 ​× ​10^5^	1.93 ​× ​10^7^	3.15 ​× ​10^5^
Colon	5.48 ​× ​10^4^	7.68 ​× ​10^4^	1.13 ​× ​10^5^
Duodenum	2.77 ​× ​10^4^	1.86 ​× ​10^6^	1.36 ​× ​10^6^
Serum	9.52 ​× ​10^7^	9.19 ​× ​10^7^	n.c.

a The analysis was done in technical duplicate per tissue and values represent the mean. The viral loads are *VP60* gene copies per μg of total RNA.

b n.c.: sample not collected.

In summary, we reported and identified one RHD case caused by the GI.2 strain, a new RHDV variant, in China. Subsequently, we amplified and sequenced the complete genome of the SC20-01 strain and found that the homologies of ORF1 between the SC20-01 and the Germany isolate (MN901451) were greater than 99%; the identity of ORF2 were as high to 100%. Moreover, in our animal experiment study, we demonstrated that GI.2 can replicate efficiently in the reproductive system, which has never been reported before.

In previous studies, the main target organs of GI.1 were the liver, lung, and spleen. Beyond that, the virus can also be detected in the trachea, kidneys, digestive tract, muscles, and central nervous system (Kimura et al., 2001; Gall et al., 2007). However, GI.2 expands the tissue distribution to the reproductive system, which has never been reported in GI.1-positive rabbits (Neimanis A. et al., 2018). In our study, we detected the virus in the gonads of infected female rabbits and demonstrated that GI.2 can replicate efficiently in the reproductive system. This has not yet been described for RHD and may provide additional insight into its pathogenesis.

Through sequence alignment analysis, we found that the SC20-01 strain was closely related to the prevalent European GI.2 strain and that both of these strains had the same recombination events. Thus, we suspected that the Chinese GI.2 strain might have come from Europe. However, there is no GI.2 epidemic reported in China's surrounding areas, which raises the question of how it was imported into the interior of China. Through communication with local farm staff and veterinarians, we learned that most of the breeding rabbits' semen in Sichuan Province was imported from Europe under the classification of pig semen because rabbit semen is not included in the import license list of China customs. Because RHDV is not in the test items in imported pig semen, the contaminated samples may spread RHDV quickly when large-scale artificial insemination takes place. To prove this hypothesis, we randomly selected ten tubes of commercial rabbit semen at a market near the outbreak farm and detected the RHDV with *VP60* specific primers. Seven of the samples were positive, and the sequencing results indicated that the amino acid was almost the same as in SC20-01 (data not shown). Combined with the phenomenon that virus can be detected in the reproductive organs, such as the testes and uteri of dead rabbits. This strongly suggests that GI.2 might be vertically transmitted from female rabbits to young rabbits via semen.

The outbreak of RHDV GI.2 in China has once again proved that RHDV is a highly contagious and fatal virus. Previous epidemiological data indicated that RHDV was mainly transmitted by contact or through vectors such as mosquitoes and flies. It has not been reported that RHDV can be transmitted through semen. However, the findings suggest that the outbreak of RHD in Chengdu was most likely caused by virus-contaminated semen. Of course, more experiments are needed to prove this transmission model, which will be the focus of our upcoming research.

## Footnotes

This study was supported by the Chinese Natural Sciences Foundation (No. 32172832, No. 32000109), Shanghai Sailing Program (20YF1457700), the China Postdoctoral Science Foundation (No. 2019M660885, No. 2021T140718). The authors declared that they had no competing interests. This research was approved by the Ethics Committee of Shanghai Veterinary Research Institute, Chinese Academy of Agricultural Sciences. The international guidelines for the care and use of animals have been followed. All animal experimental procedures were approved and performed in compliance with the guidelines of the Animal Research Ethics Board of Shanghai Veterinary Institute (Shanghai, China), CAAS (no. SHVRI-SZ-20200709-01). All methods were carried out following relevant guidelines and regulations. All animal handling and methods complied with the ARRIVE guidelines.

Supplementary data to this article can be found online at https://doi.org/10.1016/j.virs.2022.04.003

The following is the supplementary data related to this article:

Multimedia component 1 Multimedia component 1
