# Supplementary material for: The outbreak of rabbit hemorrhagic virus type 2 in the interior of China may be related to imported semen
Source: Virol Sin. 2022 May 2;37(4):623–6. doi: 10.1016/j.virs.2022.04.003 (PMC9437509; doi:10.1016/j.virs.2022.04.003)
Supplement: Multimedia component 1 [file mmc1.docx]

**Virologica Sinica**

**Supplementary Data**

**The outbreak of** **rabbit hemorrhagic virus type 2** **in** **the interior of China may be related to imported** **semen**

Ruibin Qi ^a, 1^, Chunchun Meng ^a, 1^, Jie Zhu ^a, 1^, Hang Li ^a^, Qiuhong Miao ^a^, Jingyu Tang ^a^, Aoxing Tang ^a^, Hongyuan Guo ^a^, Chuncao Liu ^a^, Chuanfeng Li ^a^, Zongyan Chen ^a^, Fang Wang ^b^, Qinwen Zhang ^c, *^, Guangqing Liu ^a, *^

^a^ Innovation Team of Small Animal Infectious Disease, Shanghai Veterinary Research Institute, Chinese Academy of Agricultural Sciences, Shanghai, 200241, China

^b^ Institute of Veterinary Medicine, Jiangsu Academy of Agricultural Sciences, Nanjing, 210014, China

^c^ Veterinary Medicine Department of Agricultural and Animal College, Qinghai University, Xining, 810016, China

^*^Corresponding author

E-mail addresses: liugq@shvri.ac.cn (G. Liu); zqw_runner@163.com (Q. Zhang)

^1^ Ruibin Qi, Chunchun Meng, and Jie Zhu contributed equally to this work.

**Supplementary Materials**

**Materials and methods**

***Hemagglutination (HA) assay***

The livers of the dead rabbits were homogenized with 0.1 mol/L phosphate buffered saline (PBS, pH = 7.5) and centrifuged at 10,000 *×g* for 30 min to collect the supernatants. A hemagglutination assay (HA) test was performed using these supernatants and 1% human erythrocytes of blood group O. Then, 50 μL PBS was added to wells 1–11 on the microtiter plate, and an equal volume of liver supernatants was added to the 12th well. Subsequently, 50 μL liver supernatants were added to the first well and mixed with a micropipettor. Then, 50 μL mixture were added to the second well, and two-fold dilutions were added to wells 1–11. Finally, 50 μL of 1% human type O erythrocytes were added to every well and mixed gently. The HA point titers were determined after incubation at 25 °C for 1 h.

***Transmission electron microscopy (TEM) detection***

The liver supernatants of dead rabbits were clarified with 30% sucrose solution and washed with PBS to remove sucrose. The purified material was negatively stained with 1% uranyl acetate (pH = 4.5) and analyzed with TEM (H-7500, Hitachi, Japan) operating at 80 kV.

***Reverse Transcription-Polymerase Chain Reaction (RT-PCR)***

Total RNA was extracted from the livers of dead rabbits with TRIzol Reagent (Invitrogen, Boston, USA), according to the manufacturer’s protocol. Each RNA sample was reverse-transcribed to cDNA with M-MLV Reverse Transcriptase (Promega, Madison, USA). Primers, which are used to amplify the full-length genome sequence of SC20-01 isolate, were designed according to the sequence of the JX/CHA/97 strain (DQ205345) (Liu et al., 2006). The PCR products were cloned with pMD18-T and sequenced.

***Phylogenetic analysis of RHDV isolates***

In this study, total of 21 and 19 representative genome sequences of GI.2 and GI.1 endemic strains, respectively, from Europe and North America were selected randomly and downloaded from GenBank. Three phylogenetic trees based on the *VP60* gene as well as structural protein-coding region (nt 5305–7378) and non-structural protein-coding region (nt 10–5304) sequences of RHDV were inferred through the maximum likelihood method with 1000 replicates using MEGA5.0 software. Three genome sequences of rabbit calicivirus (RCV) were also chosen as the out-groups.

***Recombination detection***

After selecting 324 genome sequences of RHDV and three genome sequences of RCV from GenBank, the recombination detection program (RDP) v4.56 software package was used to detect the recombination of the SC20-01 strain with the BOOTSCAN, GENECONV, Maximum Chi Square (MAXCHI), RDP, and Sister Scanning (SISCAN) methods. The *P*-value cutoff was set at 0.05 throughout,

***Divergence scan***

The Simplot program was used to assess the divergence of the nucleotide sequences within the SC20-01 strain. CBAnd1 (KP090976) and P19 (KY765610) were used as putative parental strains, and JX/CHA/97 (DQ205345) was used as a control strain. A sliding window (200 nt) was moved along the entire coding sequence, in steps of 20 nucleotides. The reliability of the recombination sites was assessed using a genetic algorithm for recombination detection (GARD, <http://www.datamonkey.org>).

***Experimental design of the animal study***

To confirm the infection of the SC20-01 strain, six 6-week-old SPF rabbits were randomly divided into two groups of three and injected with 1 mL of the supernatants of homogenized livers or PBS; then observed for three days. All animal experimental procedures were approved and performed in compliance with the guidelines of the Animal Research Ethics Board of Shanghai Veterinary Institute (Shanghai, China), CAAS (no. SHVRI-SZ-20200709-01).

***Quantification of viral copies***

Total RNA was extracted from different tissues, including liver, cholecyst, lung, spleen, heart, kidney, gonad, urine, pancreas, brain, trachea, colon, duodenum, and serum, as previously described. The viral genome load (*VP60* gene copies per μg of total RNA) in different tissues was determined using the SYBR-Green I real-time RT-PCR method.

**Table S1 RHDV and RCV sequences used in this study**

| **Strain** | **GenBank**  **accession number** | **Year of collection** | **Country** | **Strain** | **GenBank**  **accession number** | **Year of collection** | **Country** |
| --- | --- | --- | --- | --- | --- | --- | --- |
| **RHDV GI.1** |  |  |  | **RHDV GI.2** |  |  |  |
| UT-01 | EU003582 | 2001 | USA | AUS/VIC/AWA-1/2016 | MF421691 | 2016 | Australia |
| IN-05 | EU003578 | 2005 | USA | RED2016 | MG602006 | 2016 | Poland |
| JX/CHA/97 | DQ205345 | 1997 | China | CBMad17-3 | MF407657 | 2017 | Portugal |
| NZL/Canterbury/Lincoln/517/2013 | KT006721 | 2013 | New Zealand | Zar06-12 | KP129399 | - | Spain |
| Turretfield 09 South Australia | KF594476 | 2009 | Australia | AUS/WA/PTH-3/2016 | MF421698 | 2016 | Australia |
| AUS/SA/BillaKallina/2004 | KT006740 | 2004 | Australia | AUS/VIC/DUN-2/2016 | MF421687 | 2016 | Australia |
| AUS/SA/FlindersRanges/2002 | KT006737 | 2002 | Australia | AUS/VIC/AC-1/2016 | MF421684 | 2016 | Australia |
| AUS/ACT/AIN-1/2009 | KX357653 | 2009 | Australia | BLMT-1 | KT280060 | 2015 | Australia |
| AUS/NSW/OUR-1/2014/06 | KY628318 | 2014 | Australia | AUS/NSW/CAR-3/2016 | MF598302 | 2016 | Australia |
| AUS/TAS/SouthArm/2007 | KT006743 | 2007 | Australia | RHDV2-NL2016 | MN061492 | 2016 | Netherlands |
| ZD0 | KU882095 | 2000 | Poland | Bremerhaven-17 | MN901451 | 2017 | Germany |
| GSK | KU882092 | 1998 | Poland | VMS | MG602007 | 2017 | Poland |
| Ascot | EF558575 | - | UK | CBMert14-2 | KM115713 | 2014 | Portugal |
| SD | Z29514 | - | - | Zar11-11 | KP129398 | 2010 | Spain |
| P19 | KY765610 | 1994 | Portugal | CBAnd1 | KP090976 | 2012 | Spain |
| Saudi Arabia | DQ189078 | - | Saudi Arabia | RHDV-N11 | KM878681 | 2011 | Spain |
| NZ54 | EF558579 | - | New Zealand | Seg08-12 | KP129396 | - | Spain |
| Czech strain V351 | U54983 | - | Czekh | Algarve1 | KF442961 | 2013 | Portugal |
| FRG | NC_001543 | - | - | CBMora14-1 | KM115683 | - | Portugal |
| **RCV** |  |  |  | CBMad17-1 | MF407655 | 2017 | Portugal |
| MIC-07 | EU871528 | 2007 | Australia | CBVal16 | KM979445 | 2012 | Portugal |
| MIC-07 | NC_011704 | 2007 | Australia |  |  |  |  |
| AUS/ACT/Gudg-26/2013 | KX357655 | 2013 | Australia |  |  |  |  |
